# Pharmacokinetics of 7,8-dihydroxyflavone in neonatal mice with hypoxia-ischemia related brain injury

**DOI:** 10.3389/fphar.2024.1508696

**Published:** 2025-01-15

**Authors:** Sin Yin Lim, Cameron O. Scarlett, Sefer Yapici, Peter Ferrazzano, Pelin Cengiz

**Affiliations:** ^1^ Pharmacy Practice and Translational Research Division, School of Pharmacy, University of Wisconsin-Madison, Madison, WI, United States; ^2^ Analytical Instrumentation Center, School of Pharmacy, University of Wisconsin-Madison, Madison, WI, United States; ^3^ Waisman Center, School of Medicine and Public Health, University of Wisconsin-Madison, Madison, WI, United States; ^4^ Department of Pediatrics, School of Medicine and Public Health, University of Wisconsin-Madison, Madison, WI, United States

**Keywords:** pharmacokinetics, 7,8-dihydroxyflavone, hypoxia-ischemia, neonate, tyrosine kinase B

## Abstract

**Introduction:**

7,8-Dihydroxyflavone (7,8-DHF) is a promising translational therapy in several brain injury models, including the neonatal hypoxia-ischemia (HI) model in mice. However, the neuroprotective effect of 7,8-DHF was only observed in female, but not male, neonatal mice with HI brain injury. It is unknown whether HI-induced physiological changes affect brain distribution of 7,8-DHF differently for male versus female mice. We aimed to evaluate the impact of sex on the pharmacokinetics of 7,8-DHF in plasma and brain neonatal mice following experimentally induced HI brain injury.

**Methods:**

Left-sided HI brain injury was induced in postnatal day 9 (P9) mice, followed by a 5 mg/kg intraperitoneal injection of 7,8-DHF. A liquid chromatography-tandem mass spectrometry method was developed to quantitate the drug concentration in plasma samples, as well as in samples from the left and right brain hemispheres. A nonlinear mixed-effects model was used to analyze the plasma and brain concentration-time data. A semi-quantitative approach was used to evaluate the concentrations of two active O-methylated metabolites of 7,8-DHF (8H7M-flavone and 7H8M-flavone) in both plasma and brain samples.

**Results:**

Our PK analyses show that plasma 7,8-DHF concentrations followed a two-compartment PK model, with more than 95% eliminated by 3 h after the IP injection. Sex was not significantly associated with the PK of 7,8-DHF; however, HI brain injury was associated with a 21% reduction in clearance (p < 0.01). The distribution of 7,8-DHF to the brain was rapid; however, the extent of brain distribution was low with the right and left brain-to-plasma partition coefficients being 8.6% and 9.9%, respectively. Additionally, both O-methylated metabolites of 7,8-DHF were detected in the plasma and brain.

**Conclusion:**

The plasma and brain PK of 7,8-DHF in neonatal mice were similar between males and females. The low extent of 7,8-DHF brain distribution and the potential effects of the active metabolites should be considered in future studies evaluating the therapeutic effects of 7,8-DHF.

## 1 Introduction

Perinatal hypoxia-ischemia (HI) related brain injury results in death and neurologic disabilities in thousands of neonates and children every year in the US. (Douglas-Escobar and Weiss, 2015; Kurinczuk et al., 2010) Since 2007, therapeutic hypothermia (TH) has become the standard of care for infants ≥36 weeks gestation with moderate to severe hypoxic-ischemic encephalopathy (HIE) in high-resource countries. However, treatment needs to be started within 6 hours, and even then, half of the treated neonates still die or endure neurologic disability (Lee et al., 2013; Shankaran et al., 2017; Wood et al., 2016; Shipley et al., 2021). Furthermore, the effectiveness and safety of TH in low- to middle-income countries remain controversial, as recent trials suggested that TH did not significantly reduce death and moderate/severe disability at 18–22 months (Krishnan et al., 2021; Bandya et al., 2017). Consequently, there is an urgent need for additional therapeutic strategies to be tested in both normothermic and hypothermic neonates in order to improve HIE outcomes globally.

Various experimental adjuvant therapies are currently under investigation in both clinical and pre-clinical settings to complement TH, such as erythropoietin, xenon, stem cells, melatonin and others (Molloy et al., 2023; Zhou et al., 2020). Over the last decade, a small molecule ligand to tyrosine kinase B receptors (TrkB) called 7,8-dihydroxyflavone (7,8-DHF) has been extensively studied in different animal models for its neuroprotective properties (Cikla et al., 2016; Liu et al., 2016; Wang et al., 2024). 7,8-DHF mimics the activities of the neurotrophin brain-derived neurotrophic factor and activates TrkB, resulting in neuronal differentiation and survival. 7,8-DHF exhibits promising benefits in various neurological diseases such as Parkinson’s, Alzheimer’s, traumatic brain injury, hypoxia-ischemia, stroke, and depression (Liu et al., 2016; Wang et al., 2024; Lim and Cengiz, 2022).

In an adult mouse middle cerebral artery occlusion model of stroke, 7,8-DHF reduces infarct volumes and in a kainite mouse model of epilepsy reduces neuronal apoptosis (Jang et al., 2010). Similarly, we have previously reported that 7,8-DHF increases hippocampal neuronal survival by increasing phosphorylated TrkB following *in vivo* HI in neonatal mice and *in vitro* in hippocampal sexed neurons (Cikla et al., 2016; Chanana et al., 2024a; Chanana et al., 2024b). Interestingly, this short- and long-term neuroprotective effect of 7,8-DHF is only observed in females, but not in male neonatal mice. This sex-specific 7,8-DHF dependent neuroprotection requires the presence and upregulation of estrogen receptor-α (Cikla et al., 2016). These findings are interesting because of the clinical observation that female neonates have better HI-related neurological outcomes compared with males (Rosenkrantz et al., 2019; Wood T. R. et al., 2020). However, given the sex differences in cerebrovascular development and barrier genesis (Collignon et al., 2024), it raises the question of whether HI-induced physiological changes affect brain distribution of 7,8-DHF differently for males versus females, further contributing to the observed sex differences in the effects of 7,8-DHF in neonatal mice.

The pharmacokinetics (PK) of 7,8-DHF have been studied using *in vitro* assays and in adult animals (Liu et al., 2016). 7,8-DHF is rapidly absorbed orally and can penetrate the blood-brain barrier (BBB). 7,8-DHF appears to be a substrate of influx and efflux transporters, such as P-glycoprotein (P-gp) and organic anion transporting polypeptides (OATPs), which may affect its absorption and disposition (Chen Y. et al., 2019). 7,8-DHF binds to human serum albumin; its binding to other plasma proteins, such as alpha-1 acid glycoprotein, is unknown (Fliszár-Nyúl et al., 2019). In adult male mice, 7,8-DHF was found to have a short half-life of 2.2 h (Liu et al., 2013). Its elimination appears to occur primarily through glucuronidation, sulfation, and methylation, rather than metabolism by cytochrome P450 enzymes (Liu et al., 2013; Sun et al., 2017). Due to the developmental changes in physiology and anatomy, these pharmacokinetic data may not extrapolate to neonatal mice. Furthermore, it is unknown whether HI-induced physiological changes of the brain affect the drug distribution of 7,8-DHF differently in male versus female neonatal mice, which may contribute to sex differences in 7,8-DHF neuroprotective effects. To further understand the sex-specific effects of 7,8-DHF in HI-related neonatal brain injury, we reported the plasma and brain PK of 7,8-DHF in postnatal day 9 (P9) mice following experimentally induced HI brain injury, and evaluated potential sex-specific differences in 7,8-DHF brain distribution.

## 2 Materials and methods

### 2.1 Drugs and reagent

7,8-DHF hydrate was purchased from Sigma-Aldrich (St. Louis, MO). Deuterated internal standard (D4-genistein) was purchased from C/D/N isotopes (Pointe-Claire, Quebec, CA). Ammonium bicarbonate was purchased from Sigma-Aldrich (St. Louis MO). Acetonitrile and water solvents for LCMS analysis were Optima LC/MS grade (Fisher Chemicals Fairlawn, NJ). All stocks and internal standard were dissolved in DMSO (Sigma-Aldrich St. Louis, MO).

### 2.2 Animals

All procedures on mice were carried out in adherence with NIH Guide for the Care and Use of Laboratory Animals using protocols reviewed by the Institutional Animal Care and Use Committee at University of Wisconsin-Madison.

Adult male and female C57BL/6 mice (4–6 weeks old) were purchased from Jackson Laboratory (Bar Harbor, ME), used as breeders, and sacrificed at 6 months of age. Mice were housed in ventilated cages, on a 12-h light/dark cycle with food and water available *ad libitum*. P9 neonatal mice from the adult breeding pairs were used in the experiments. A unilateral HI brain injury was induced in P9 mice as previously described in detail (Chanana et al., 2024b; Vannucci and Vannucci, 2005). Briefly, the left common carotid artery was exposed and electro-cauterized, followed by a 2-h recovery in their dams. The mice were operated on a heated pad followed by recovery in a heated chamber at 36°C. They were then exposed to 10% oxygen (balanced nitrogen) in a hypoxia chamber for 50 min at 36°C to induce a unilateral hypoxic-ischemic insult. The sham-operated mice underwent the same procedure except without electro-cauterization of left common carotid artery and exposure to 10% oxygen. After the induction of HI, the mice were placed in the cages with their dams. Core temperatures of mice were not monitored.

#### 2.2.1 Dosing and sample collection

Since the original neuroprotective effects are reported in literature for the first time by Jang et al. (2010), 7,8-DHF has been studied in small and large animal models of disease at the 5 mg/kg (intraperitoneal [IP] or subcutaneous) doses (Emili et al., 2022). We have also previously showed that this dose was neuroprotective in female neonatal mice post-HI (Cikla et al., 2016; Chanana et al., 2024a; Chanana et al., 2024b). Thus, a dose of 7,8-DHF (5 mg/kg), diluted in 0.01 M PBS, was administered via IP injection 10 min following hypoxia. They were sacrificed via cardiac puncture at 10 min, 30 min, 1 h, and 3 h post- 7,8-DHF injection. At each timepoint, there were 5 female and 5 male HI mice and 5 female and 5 male sham mice. A single blood sample, along with the right and left brain hemispheres, was collected from each mouse. Blood samples were collected in EDTA tubes, and plasma was obtained by centrifugation of the blood for 3 min at 2,200 × *g* at 4°C. The whole brain was dissected out and the right and left brain hemispheres were separated. The brain hemispheres were then rinsed with chilled PBS and frozen in liquid nitrogen. Plasma and brain samples were stored in −80°C until bioanalysis.

### 2.3 Bioanalytical method development and validation

Plasma and brain concentrations of 7,8-DHF were measured using liquid chromatography-tandem mass spectrometry (LC/MS/MS). Calibrators and quality controls (QCs) were prepared by diluting stock solutions of 7,8-DHF in blank pooled mouse plasma (Innovative Research Novi, MI) or blank brain extracts from untreated animals. Plasma samples, calibrators, and QCs were prepared for LC/MS/MS analysis using Ostro phospholipid removal 96-well plates (Waters, Milford, MA). Briefly, 50 µL of plasma was placed in the Ostro plate assembled on a 2-mL receiver plate. For each sample 150 µL of acetonitrile/0.1% formic acid solvent containing the D4-genistein internal standard (ISTD) was added and mixed using a wide-mouth pipet on a 12-channel pipet. After a 2-min incubation the sample was pushed through the plate using a Waters positive-pressure manifold or centrifugation. After filtration samples were dried under nitrogen (∼30 min) and then resuspended in 25% acetonitrile/0.1% formic acid to reduce organic solvent concentration for LC retention. Processing blank samples with this method and spiking post-processing at low, mid and high QC levels gave analyte concentrations of 102% of theoretical showing good percent recovery.

The concentration of 7,8-DHF in brain samples was measured after tissue homogenization in 150 mM ammonium bicarbonate. Briefly, frozen tissue from treated P9 animals or blank adult brains were weighed in homogenization tubes (Omni/ThemoFisher, Waltham, MA) pre-loaded with 1.4 mm ceramic beads. Ammonium bicarbonate (2 mL/g tissue) was added, and samples were then homogenized using a 48-place reciprocating bead-mill homogenizer (Omni, ThermoFisher Waltham, MA) using the “Brain” setting and stored on ice. Calibrators and QC samples (60 µL) were spiked with 25x concentrated stock solutions. Similar to the plasma samples brain samples, calibrators and QCs were processed by phospholipid removal on Ostro plates after protein precipitation.

Samples were analyzed by triplicate LC/MS/MS injections on a Waters I-class UPLC (Milford, MA) system coupled to a Sciex QTrap 5500 system (Framingham, MA) in positive electrospray mode. Samples (6.5 µL) were injected on a 2.1 × 50 mm BEH-C18 column held at 28°C. Analytes and ISTD were eluted from the column with a gradient of water/0.1% formic acid (solvent A) and acetonitrile/0.1% formic acid (solvent B) at a flow rate of 0.4 mL/min. The gradient began at 30% B and increased to 62.5% B at 1.5 min. At 1.65 min the gradient reached 95% and was held for 0.45 min. The gradient then was adjusted back to 30% B in 0.15 min and held there to re-equilibrate the column. The spectrometer was operated in MRM mode using a period method. For the first 1.25 min the spectrometer targeted 7,8-DHF (parent 255.0; products 129.1, 107.0, and 227.0) with a 50 ms dwell time for each transition. Voltages were IS 3000V; DP 156V. The collision gas was set to “high” with curtain and ion source gases set to 35. Source temperature for 7,8-DHF was 450°C. Period 2 (1.25–2.5 min) targeted D4-genistein (parent 275.0; products 154.1, 219.0, and 94.0) and O-methylated-DHF (parent 269.1; products 152.0, 226.0, and 254.0). Voltages in this period were IS 3800V; DP 151V. The collision gas was set to “high” while the other gases were set to 30 for CG, 45 for ISG1, and 30 for ISG2.

For quantitation, area under the curve (AUC) for each transition as well as a summed transition (total) AUC relative to the AUC of the internal standard were modeled with a quadratic fit using 1/x^2^ weighting using MultiQuant software (Sciex Framingham, MA). Calibrators differing by >15% from their theoretical levels were eliminated from the models. Robustness of the model was judged by the R-value (>0.995) and performance of the QC samples (three of four within 15% of theoretical, QC-low 3 ng/mL, QC-mid 20 ng/mL, QC-high 60 ng/mL). Samples, calibrators and QCs with relative standard deviation (%RSD) levels >15% were not reported/used. The limit of detection (LOD) was determined at a signal-to-noise ratio of 3 and the lower limit of quantification (LLOQ) was established at a signal-to-noise ratio of 10.

The concentrations of ions corresponding to two O-methylated metabolites of 7,8-DHF (7-hydroxy-8-methoxyflavone [7H8M-flavone; RT: 1.62 min] and 8-hydroxy-7-methoxyflavone [8H7M-flavone; RT: 1.45 min]) were evaluated using a semi-quantitative approach. The precursor ions of m/z 269.1 with a loss of m/z 15 during transition were monitored for these metabolites. As the metabolite standards are not commercially available, we quantitated the concentrations of the two metabolites using the 7,8-DHF calibration curve. The area ratio of these peaks to the ISTD were used to estimate their concentrations. Though this method of metabolite quantitation has been shown to have large fold errors (Dahal et al., 2011; Blanz et al., 2017), the purpose of the semi-quantitation was to evaluate the presence and the trends of the changes of metabolite concentrations.

### 2.4 Pharmacokinetic analysis and model development

Plasma concentration-time data of 7,8-DHF were analyzed using a nonlinear mixed-effects (NLME) modeling approach in Phoenix NLME (version 8.3; Certara USA, Inc., Princeton, NJ). The NLME approach estimates the mean PK parameters, as well as the associated intra- and interindividual variability. In our study, only one sample was collected from each animal, therefore the intraindividual variability is not discernible from the interindividual variability. Despite the limitation, the NLME approach was able to provide good estimation of the PK parameters and covariate effects (Ette et al., 1995; Hing et al., 2001). We fixed the intraindividual variability to the average bioanalytical variability based on QC samples (coefficient of variation [CV] of 5% for plasma and 10% for brain samples) and allowed the model to estimate the interindividual variability. Fixing the intraindividual variability at different values may impact the parameter estimation; further discussion is presented in the Supplementary Material. The Laplacian method was used as the maximum likelihood estimation algorithm. Model selection was based on the Akaike information criterion (AIC), objective function (−2 log- likelihood, −2LL), diagnostic plots, and parameter estimation uncertainty. The concentration values below LLOQ were censored and treated as any value less than the LLOQ value. The final PK model performance was evaluated using bootstrap (n = 1,000) and visual predictive checks (n = 1,000). Additional information about the model development is available in the Supplementary Material.

**FIGURE 1:**
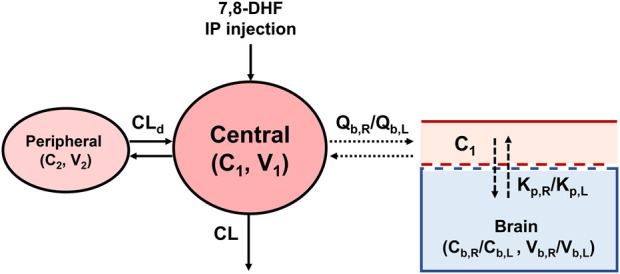
The 7,8-DHF plasma and brain pharmacokinetic model scheme.

A 2-compartment model was selected as the base PK model based on AIC values and diagnostic plots (Figure 1). The model is described by the following differential equations:

$$V_{1}\cdot \frac{dC_{1}}{dt}=input-CL_{d}\cdot \left(C_{1}-C_{2}\right)-CL\cdot C_{1}$$

$$V_{2}\cdot \frac{dC_{2}}{dt}=CL_{d}\cdot \left(C_{1}-C_{2}\right)$$

where *input* is the amount of 7,8-DHF given via IP injection, which is assumed to have immediate absorption, resembling an intravenous injection. *C*_*1*_ and *C*_*2*_ are the 7,8-DHF concentration in the central and peripheral compartments, respectively. *V*_*1*_ and *V*_*2*_ are the volume of distribution in the central and peripheral compartments, respectively. *CL* represents the clearance of 7,8-DHF. *CL*_*d*_ denotes the distributional clearance between the central and peripheral compartments. Because the bioavailability of 7,8-DHF given by IP injection is unknown, the PK parameters from our model are interpreted as “apparent” parameters, which may be different if the drug is given by a different route. Interindividual variability using the exponential model was estimated for CL; interindividual variabilities for other PK parameters had a high shrinkage (>30%) and were removed from the model. Sex (male versus female) and surgical treatment (HI versus sham) were evaluated as a covariate for CL using the stepwise covariate search, which employed likelihood ratio tests. Additional derived parameters were calculated. The terminal half-life was determined based on the terminal slope, which was obtained by converting the micro-constants from the 2-compartment model to macro constants (Talevi and Bellera, 2021) The area under the concentration-time curve from 0 to 3 h (AUC_0–3hr_) was obtained by integrating the curve over this time period, while the total AUC (AUC_0-∞_) was derived using the dose divided by clearance.

To estimate the distribution of 7,8-DHF to right and left brain hemispheres, a sequential modeling approach was used. First, the *post hoc* individual PK parameters estimated from plasma concentrations were used to predict the individual plasma concentrations (C_1_). C_1_ was then used to drive the distribution of 7,8-DHF to the brain using a physiologically based PK model with perfusion-limited distribution (Figure 1). The perfusion-limited distribution model well describes drugs with rapid tissue distribution (Jones and Rowland-Yeo, 2013). The model is selected based on the physicochemical properties of 7,8-DHF (low molecular weight, 254.2 g/mol; lipophilic, logP 3.3; small surface polar area, 66.8 Å^2^) and the rapid brain distribution observed in adult mice after an oral 7,8-DHF dose (Liu et al., 2013; National Center for Biotechnology Information, 2024; Pardridge, 2012). The brain PK model is described by the following differential equations:

$$V_{b,R}\cdot \frac{dC_{b,R}}{dt}=Q_{b,R}\cdot \left(C_{1}-\frac{C_{b,R}}{K_{p,R}}\right)$$

$$V_{b,L}\cdot \frac{dC_{b,L}}{dt}=Q_{b,L}\cdot \left(C_{1}-\frac{C_{b,L}}{K_{p,L}}\right)$$

$$K_{p,L}=K_{p,R}\times R_{kp}$$

where *V*_*b,R*_ and *V*_*b,L*_ represent the right and left brain volume, respectively. These volumes have been previously reported to constitute 2.5% of body weight in neonatal mice and are consistent across both males and females (Wang et al., 2017) *Q*_*b,R*_ and *Q*_*b,L*_ depict the cerebral blood flow to the right and left brain hemisphere, respectively. Cerebral blood flow in neonatal mice has been reported to be 54 mL/100 g/min (Ek et al., 2015) The reported physiological values for brain volume and cerebral blood flow were used as constants in our model. *C*_*1*_ is the plasma concentration and the driver of 7,8-DHF brain distribution. *C*_*b,R*_ and *C*_*b,L*_ are the right and left brain hemisphere concentrations, respectively. *C*_*b,R*_ were used to estimate *K*_*p,R*_ value, which represents the right brain hemisphere-to-plasma partition coefficient. *C*_*b,L*_ were used to estimate the fold difference (denoted as *R*_*kp*_) between the right and left brain-to-plasma partition coefficients, *K*_*p,R*_ and *K*_*p,L*_. Interindividual variability using the exponential model was estimated for *K*_*p,R*_ and *R*_*kp*_, and sex and surgical treatment were tested in the covariate analysis using likelihood ratio tests.

The impact of residual blood in the brain on 7,8-DHF brain distribution was evaluated based on the simplified residual blood correction model by Friden and colleagues (Fridén et al., 2010):

$$Corrected\;C_{b}=\frac{C_{b}-V_{app}\times C_{p}}{1-V_{plasma,br}}$$

To correct for the 7,8-DHF brain concentration (*corrected C*_*b*_; ng/g of brain tissue), the amount of 7,8-DHF in the plasma within the brain was subtracted from the total amount of 7,8-DHF measured in the brain sample. *C*_*b*_ (ng/g of brain tissue) is the measured concentration in the brain, while *Cp* (ng/mL) is the measured concentration in plasma. *V*_*plasma,br*_ is the physiological plasma volume (mL/g of brain tissue) within the brain, which is about 1.26% of the brain weight of neonatal mice (Ek et al., 2015). *V*_*app*_ is the apparent volume of distribution of 7,8-DHF in plasma within the brain and it depends on the protein binding of the drug in plasma. When protein binding is low, V_app_ approaches V_plasma,br_; when protein binding approaches 100%, V_app_ is 20% lower (based on rat data) than V_plasma,br_ (Fridén et al., 2010). This means that, for a highly bound drug, the drug amount in the plasma within the brain will be 20% lower compared with the drug amount in the same volume of plasma in circulation. The protein binding of 7,8-DHF in neonatal mice is unknown. Using human serum albumin protein binding assay, 7,8-DHF appears to be highly protein bound (Fliszár-Nyúl et al., 2019). We calculated the corrected C_b_ and compared the brain-to-plasma partition coefficient using both extreme cases of protein binding (0% and 100%). The 7,8-DHF erythrocyte distribution is unknown, and erythrocyte distribution likely has little contribution to the brain concentration correction (Fridén et al., 2010); therefore, erythrocyte distribution was not considered in the correction.

### 2.5 Exploratory analysis of 7,8-DHF metabolites

The semi-quantitative plasma and brain O-methylated 7,8-DHF metabolite concentrations were summarized using descriptive statistics (mean and standard deviation) at each time point. The changes of these concentrations over time were compared with the PK profile of 7,8-DHF. Due to the exploratory nature of the analysis, no statistical analysis or PK modeling was performed on metabolite data.

## 3 Results

### 3.1 Bioanalytical analysis method development and validation

**FIGURE 2:**
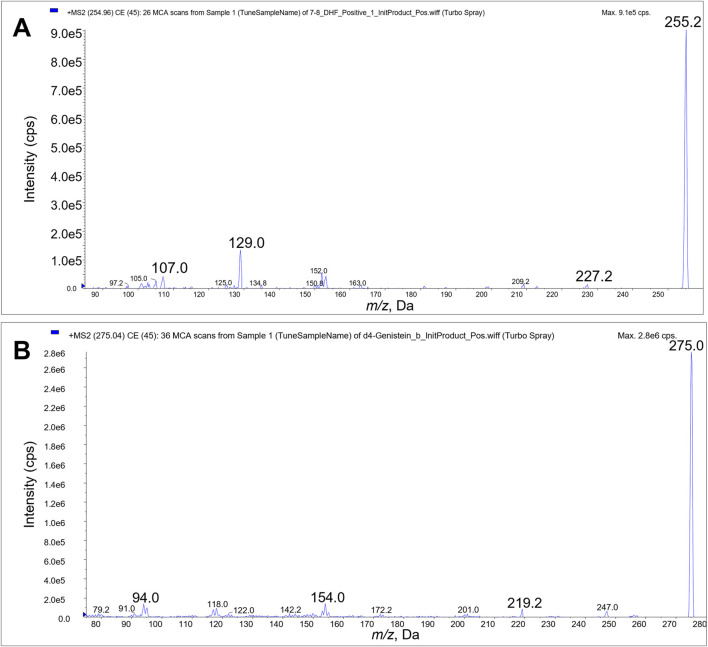
Product ion scans. Product ions 107, 129 and 227 were used for LC/MS/MS quantitation of 7,8-DHF **(A)**. Product ions 94, 154, and 219 were used for quantitation of D4-genistein internal standard **(B)**.

**FIGURE 3:**
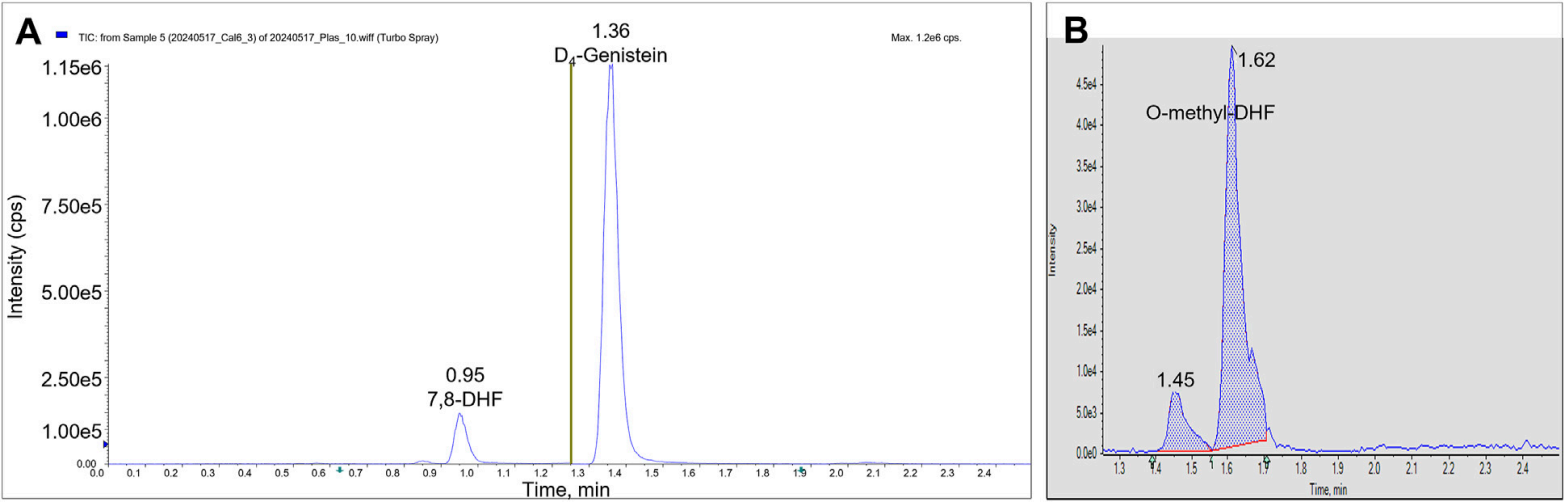
LC/MS/MS chromatograms. The total ion chromatograms from a plasma calibrator: 7,8-DHF is observed in period 1 at RT 0.95 min, and D4-genistein is seen in period 2 at RT 1.36 min **(A)**. The summed AUC chromatogram for parent ion 269, O-methyl-DHF in period 2 from a brain sample **(B)**. Two peaks are observed at 1.45 min and 1.62 min.

We identified the protonated precursors ions for 7,8-DHF and D4-genistein (ISTD) at *m/z* 255 and 275, respectively. The selected product ions of 7,8-DHF at *m/z* 107, 129, and 227 and the product ions of D4-genistein at *m/z* 94, 154, and 219 were also identified (Figure 2). The representative chromatograms for 7,8-DHF, D4-genistein, and O-methylated metabolites are shown in Figure 3. For plasma samples, the LOD and LLOQ were 0.25 ng/mL and 0.75 ng/mL, respectively. For brain samples, the LOD and LLOQ were 0.75 ng/g and 2.25 ng/g, respectively.

### 3.2 Plasma and brain pharmacokinetic model

**FIGURE 4:**
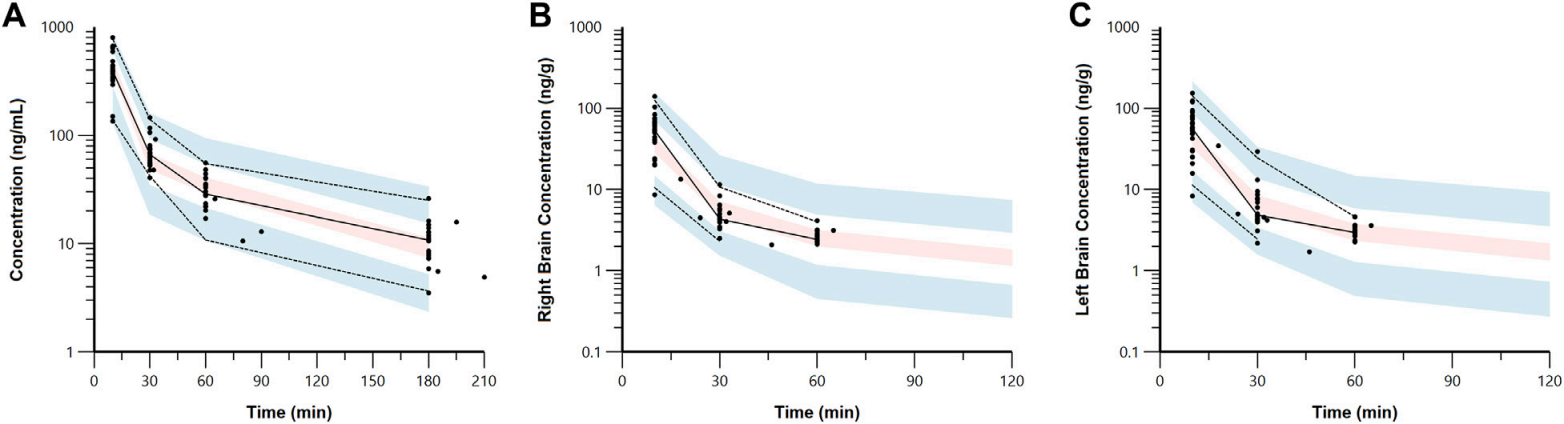
Visual predictive check plots for 7,8-DHF in plasma **(A)**, right brain hemisphere **(B)**, and left brain hemisphere **(C)** based on 1,000 simulated datasets. Black circles represent the observed data. The solid lines represent the median observed concentrations, and the dashed lines represent the 5th and 95th percentiles of the observed concentrations. The shaded areas represent the 90% confidence intervals for the model predicted 5th, 50th, and 95th percentiles.

**FIGURE 5:**
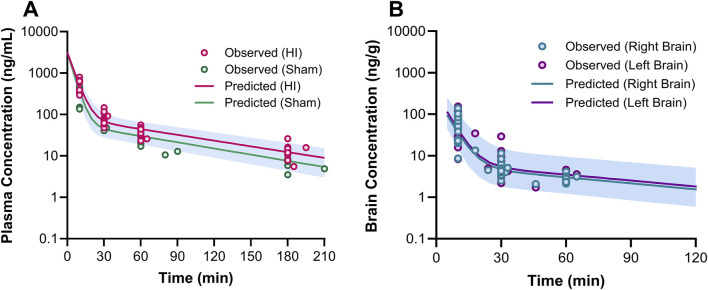
Plasma **(A)** and brain **(B)** pharmacokinetic profiles of 7,8-DHF. In graph **(A)**, the circles represent the observed concentrations for neonatal mice with hypoxia-ischemia (HI) related brain injury (red) and without brain injury (sham; green). The solid lines represent the predicted concentration-time profiles for HI (red) and sham mice (green). In graph **(B)**, the circles represent the observed right (teal) and left (purple) brain concentrations. The solid lines represent the predicted concentration-time profiles for right (teal) and left (purple) brain hemispheres.

The estimated plasma PK parameters and the results of the bootstrap analysis are summarized in Table 1. The median estimates from the bootstrap analysis were similar to the final PK parameter estimates, and the data fitting was reasonable as shown in the visual predictive check plots (Figure 4). The plasma concentrations of 7,8-DHF followed a two-compartment model where there was an initial rapid distribution occurred within 30 min of the IP injection, followed by a slower terminal phase with a terminal half-life of 1 h (Figure 5A). With the intraindividual variability fixed at 5%, the interindividual variability of clearance was moderate at 20%. For the 5 mg/kg IP injection, the estimated mean AUC by 3 h (AUC_0–3hr_) was 20.4 mg∙min/mL, which accounted for more than 95% of the estimated total AUC (AUC_0-∞_) of 21.0 mg∙min/mL. The HI treatment was statistically significantly associated with a 21% reduction in 7,8-DHF clearance (p < 0.01); sex was not significantly associated with the 7,8-DHF clearance.

**TABLE 1:** Pharmacokinetic parameter estimates.

Parameter	Population mean (RSE)	Bootstrap median (95% CI)
Plasma PK^a^		
V_1_ (mL/kg)	1,591 (48.3)	1,621 (666.5–3,035)
CL (mL/min/kg)^b^ = $\theta_{1}\times e^{\theta_{2}}$		
$\theta_{1}$	270.1 (17.7)	270.0 (182.4–338.2)
$\theta_{2}$	−0.236 (18.8)	−0.233 (−0.148–0.320)
V_2_ (mL/kg)	5,802 (34.5)	5,801 (2,901–9,831)
CL_d_ (mL/min/kg)	90.50 (29.0)	88.60 (43.54–133.9)
Proportional error (%)	5 (fixed)	—
IIV_CL_ (%)	20.3 (17.6)	19.3 (15.7–23.0)
Brain PK		
*V*_*b,R*_ and *V*_*b,L*_ (g/kg)	2.5% of body weight (fixed)	—
*Q*_*b,R*_ and *Q*_*b,L*_ (mL/min/kg)	54 mL/min/100 g brain (fixed)	—
K_p,R_	0.086 (5.37)	0.086 (0.077–0.095)
K_p,L_ ^c^ = K_p,R_ × R_kp_ R_kp_	1.158 (3.17)	1.157 (1.088–1.239)
Proportional error (%)	10 (fixed)	—
IIV_Kp,R_	46.1 (18.1)	45.8 (37.1–54.9)
IIV_Rkp_	21.9 (41.6)	21.3 (12.8–30.4)

^a^ Parameters are “apparent” values as the bioavailability of intraperitoneal 7,8-DHF injection is unknown.

^b^ Clearance of sham mice = $\theta_{1}$ (270.1 mL/min/kg) and clearance of HI mice = $\theta_{1}\times e^{\theta_{2}}$ (213.3 mL/min/kg).

^c^ K_p,L_ = 0.099.

V_1_, central volume of distribution; CL, clearance; V_2_, peripheral volume of distribution; CL_d_, distributional clearance; IIV, interindividual variability; V_b,R_ and V_b,L_, volume of right and left brain hemispheres; Q_b,R_ and Q_b,L_, cerebral blood flow to right and left brain hemispheres; K_p,R_, right brain-to-plasma partition coefficient; K_p,L_ left brain-to-plasma partition coefficient; R_kp_, left-to-right K_p_ ratio; —, not applicable.

The physiologically based brain PK with perfusion-limited distribution model well described the 7,8-DHF brain distribution (Figures 4B, C). The model parameters are shown in Table 1. The brain hemisphere volumes and cerebral blood flows were fixed to the physiological values based on previously published findings in C57BL/6 neonatal mice (Ek et al., 2015). The mean right (K_p,R_) and left (K_p,L_) brain-to-plasma partition coefficients were found to be 8.6% and 9.9%, respectively; and the partition of 7,8-DHF into the left brain was statistically significantly greater than the right brain (mean fold difference: 1.16; 95% CI: 1.09–1.23). We observed a high variability in 7,8-DHF brain distribution. With the intraindividual variability fixed at 10%, the interindividual variability of K_p,R_ was 46.1%, and the interindividual variability of the difference between right and left K_p_ values, R_kp_, was 21.9%. Overall, the concentrations in the brain were 10 times lower than those in plasma. Sex and surgical treatment were not significantly associated with the brain-to-plasma partition coefficients.

In our evaluation of the impact of residual blood on brain concentration, the corrected brain concentrations were estimated to be about 10% lower than the uncorrected brain concentrations. Protein binding had a minimal impact (∼3%) on the correction for residual blood. Using the corrected brain concentrations, the K_p,R_ was 7.6%–7.8% and the K_p,L_ was 8.9%–9.1%, slightly lower than the K_p_ values determined from the uncorrected brain concentrations (8.6% for the right brain and 9.9% for the left brain).

### 3.3 7,8-DHF metabolites in plasma and brain

**FIGURE 6:**
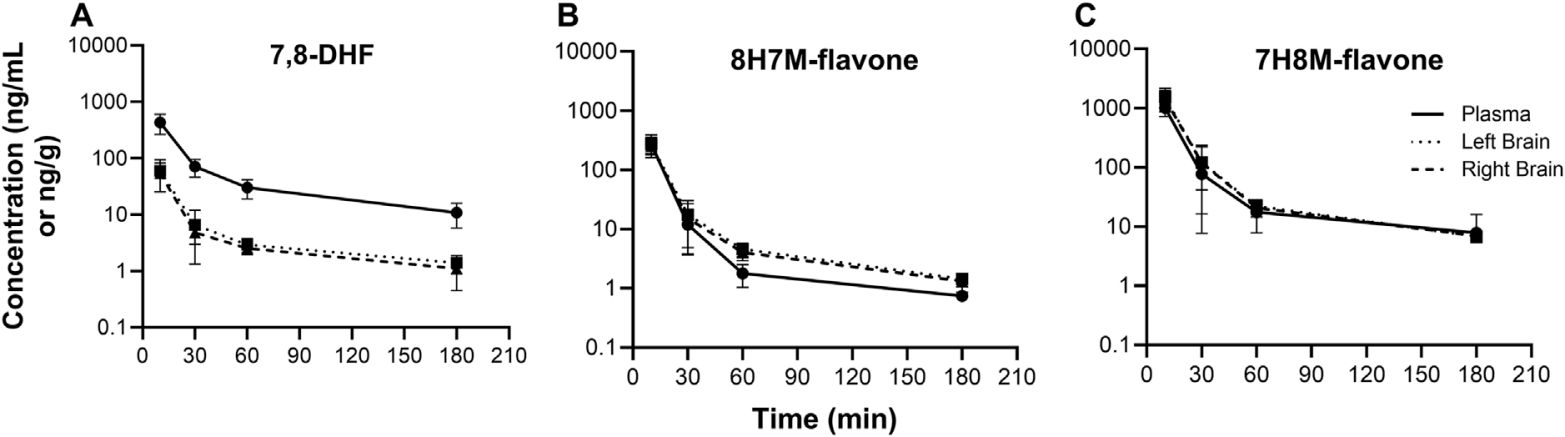
Concentration-time profiles of 7,8-DHF **(A)**, 8H7M-flavone **(B)**, and 7H8M-flavone **(C)** in plasma and brain.

**FIGURE 7:**
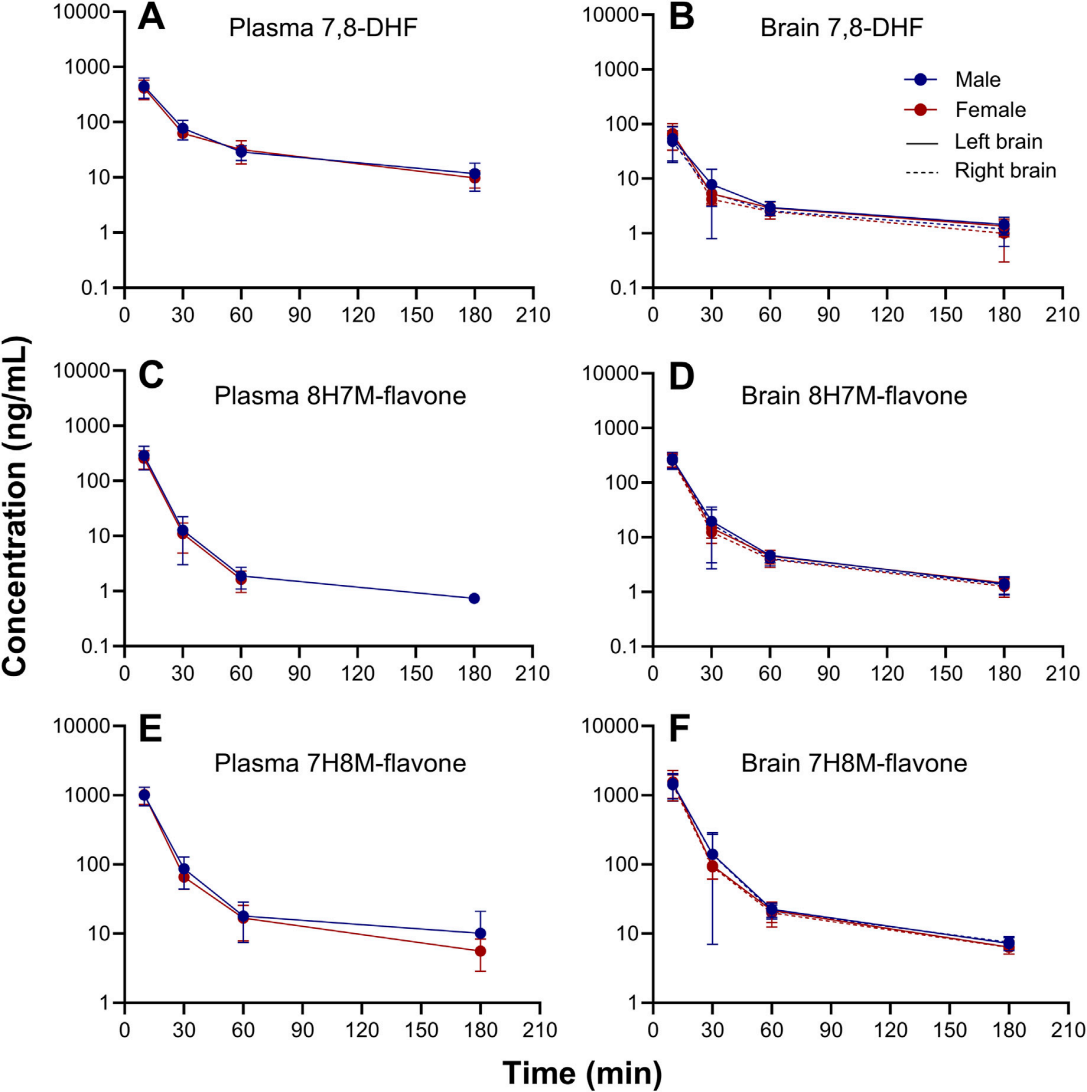
Sex-stratified concentration-time profiles of 7,8-DHF **(A, B)**, 8H7M-flavone **(C, D)**, and 7H8M-flavone **(E, F)** in plasma and brain.

O-methylated metabolites were detected in plasma and brain using the semi-quantitative approach. The changes of 7,8-DHF, 8H7M-flavone, and 7H8M-flavone concentrations over time in the plasma and brains are shown in Figure 6. It was found that the terminal half-lives were similar across these compounds. As shown above, 7,8-DHF concentrations in the plasma were 10-fold higher than those of the brains. However, the concentrations of 8H7M-flavone and 7H8M-flavone were slightly higher in the brains or similar between the plasma and brains. Like 7,8-DHF, the concentrations of O-methylated metabolites in plasma and brain did not appear to be affected by sex (Figure 7).

## 4 Discussion

We developed an LC/MS/MS method to quantitate the concentration of 7,8-DHF in neonatal mouse plasma and brain. Our PK analyses show that plasma 7,8-DHF concentrations followed a two-compartment PK model, with more than 95% eliminated by 3 h after the IP injection. Sex was not significantly associated with the PK of 7,8-DHF; however, HI brain injury was associated with a 21% reduction in clearance (p< 0.01). The distribution of 7,8-DHF to the brain was rapid; however, the extent of brain distribution was low. The right and left brain-to-plasma partition coefficients were 8.6% and 9.9%, respectively. Additionally, both O-methylated metabolites of 7,8-DHF (8H7M-flavone and 7H8M-flavone) were detected in the plasma and brain.

The PK profile of 7,8-DHF given by IP injection in our neonatal mice differs from the oral 7,8-DHF PK profile in adult mice reported by Liu and colleagues (Liu et al., 2013). After the IP injection, we observed a PK profile similar to that of a 2-compartment model with intravascular administration, characterized by an initial rapid distribution of 7,8-DHF, followed by a slower terminal phase (terminal half-life: 1 h). In adult mice given an oral dose of 50 mg/kg of 7,8-DHF, the oral absorption was rapid, with the maximum 7,8-DHF concentration achieved within 10 min (Liu et al., 2013). During the distribution phase, a second peak was observed at 120 min, followed by a terminal phase with a half-life of 2.2 h (Liu et al., 2013). A second peak could result from enterohepatic recirculation, where the excreted glucuronide metabolite in the gastrointestinal tract is converted back to the parent drug, which is subsequently reabsorbed into the circulation. Flavonoids are known to be subject to enterohepatic recirculation (Zeng et al., 2016). This may also explain the longer terminal half-life in adult mice, contrasting with the fact that neonates typically have a longer half-life due to their immature drug-eliminating organs (Lim and Pettit, 2019). Additionally, a shorter terminal half-life in neonatal mice could result from their smaller 7,8-DHF volume of distribution compared with adult mice. With lower fat content in neonatal mice, a lipophilic drug like 7,8-DHF may have a lower volume of distribution. (National Center for Biotechnology Information, 2024; Gargiulo et al., 2014). We also found that the clearance in HI-treated mice was 21% lower than in sham mice. This suggests that HI brain injury may affect the metabolism of 7,8-DHF. 7,8-DHF is a substrate of catechol-O-methyltransferase (COMT), which is expressed in the neonatal brain (Tunbridge et al., 2006). The brain COMT activity, or other metabolic pathways, may be reduced as a result of HI brain injury. We did not observe a significant difference in the brain concentration of 7,8-DHF between HI-treated and sham mice. However, the 7,8-DHF brain concentrations showed large variability, which may limit the detection of any differences.

7,8-DHF is known to penetrate the blood-brain barrier (BBB) (Jang et al., 2010). In neonatal mice, we found that the distribution of 7,8-DHF to the brain was rapid, with the maximum brain concentration occurring at the time of maximum plasma concentration. However, the partition of 7,8-DHF into the brain was low (8%–10%), with brain concentrations about 10-fold lower than those in plasma. The lower brain distribution in our study disagrees with the findings in the adult mouse study by Liu and colleagues, where brain concentrations were close to plasma concentrations (Liu et al., 2013). HI related brain injury in human neonates result in a decrease in cerebral blood flow rate and an increase in BBB permeability (Lee et al., 2017; Volpe et al., 1985). Similarly, the experimentally induced left-sided HI brain injury in neonatal mice showed similar changes in the left brain hemisphere (Ek et al., 2015). These changes can result in a slower rate of drug distribution to the brain, but an increased permeability of the BBB (Thompson and Ronaldson, 2014; Upton et al., 2000). In this study, we did not observe a difference in brain distribution in HI-treated and sham mice. However, we observed a lower extent of 7,8-DHF brain distribution, K_p_, in neonatal mice compared with adult mice. A lower K_p_ could indicate a lesser degree of 7,8-DHF accumulation in the brain due to processes like protein binding, lysosomal trapping, and lipid dissolution (Jones and Rowland-Yeo, 2013). A lower K_p_ could also indicate that drug transporters play a role in 7,8-DHF brain distribution. Using the Caco2 monolayer assay, 7,8-DHF was found to be subject to various influx and efflux transporters, including P-glycoprotein (P-gp) and organic anion transporting polypeptides (OATPs) (Chen Y. et al., 2019). The human ontogeny of drug transporters appears to vary significantly (Brouwer et al., 2015). For example, P-gp is expressed in the liver and intestines early in life, OATP2B1 is highly expressed at birth but its expression decreases over time, and OATP1B1 is minimally expressed at birth. Little is known about the transporter ontogeny in BBB. A decrease in net influx transporter activity in neonatal mice may explain the lower brain distribution of 7,8-DHF. To determine if transporters are involved in 7,8-DHF brain distribution, the unbound brain-to-plasma drug concentration (K_p,uu,brain_) ratio may need to be experimentally determined (Hammarlund-Udenaes et al., 2008). Additionally, drug distribution to the brain may be affected by residual blood within the brain. In our evaluation of the impact of residual blood on 7,8-DHF brain distribution, we found that correcting for brain concentration by removing residual blood has a small impact on K_p_ estimation, with a 10% relative difference.

Cerebral blood flow in human female preterm neonates is 18% lower than males (Baenziger et al., 1994), but it is higher in males compared with females in children and adults (Taki et al., 2011; Tegeler et al., 2013). Such difference is unknown in neonatal mice and rats. Differences in cerebral blood flow may contribute to different rates of drug distribution to the brain. In this study, however, sex did not appear to be associated with 7,8-DHF brain distribution. Despite potential differences in cerebral blood flow between sexes, the relatively high cerebral blood flow and small brain hemisphere volume (Wang et al., 2017) suggest that 7,8-DHF will be rapidly distributed to the brain and reach equilibrium between the brain and plasma. Within the physiologically plausible range of cerebral blood flow, the brain concentration of 7,8-DHF is not sensitive to changes in cerebral blood flow. As a result, the 7,8-DHF brain concentration is primarily determined by the brain-to-plasma partition coefficient. Interestingly, distribution to the left brain hemisphere was slightly higher (15% relative difference, p < 0.01) than to the right. This difference was not significantly associated with sex and HI treatment. It is unclear why there is a difference in left and right brain distribution. This could be due to a type 1 error, or the large variability in brain distribution may mask the effects of sex or HI treatment.

The elimination of 7,8-DHF has been found to be primarily through glucuronidation, sulfation, and methylation (Liu et al., 2013; Sun et al., 2017). Methylation of 7,8-DHF into O-methylated metabolites (7H8M-flavone and 8H7M-flavone) by COMT has been reported and these O-methylated metabolites appeared to have agonistic effects on TrkB receptors (Liu et al., 2016; Liu et al., 2013). Using a semi-quantitative approach, we detected both 7H8M-flavone and 8H7M-flavone in the plasma and brain. It should be noted that in this semi-quantitative approach, there are no reference standards for the metabolites (Dahal et al., 2011; Blanz et al., 2017). Therefore, it could not provide accurate concentrations of these metabolites. Additionally, COMT is ubiquitous and is found in brains and in blood. We detected a small quantity of 8H7M-flavone in calibrators and QC samples in plasma and brain, and 7H8M-flavone in brain but not in plasma. *Ex vivo* metabolism might have contributed to the quantitation of these metabolites. Despite this limitation, the profiles of the O-methylated metabolites may provide some information about their PK. For example, the metabolites and 7,8-DHF had similar half-lives, suggesting that the elimination of the metabolites rate-limited by the formation of the metabolites. This means that when the metabolites are administered on their own, their elimination is likely to be faster than that of 7,8-DHF. This implies that the active metabolites may not be ideal drugs due to their fast clearance. Additionally, the detection of these active metabolites means that when the therapeutic activity of 7,8-DHF is evaluated, the effects of the metabolites may also need to be considered.

### 4.1 Study limitations and future directions

First, the brain weight of HI-treated animals may be higher than that of controls due to cerebral edema occurring during and after the HI injury (Vannucci et al., 1993), or lower than that of controls days after the HI injury as a result of brain damage (Penny et al., 2021; Chen X. et al., 2019). In this study, we found no difference in left versus right brain weight (% of body weight) among HI-treated mice (absolute difference: 0.02%, p = 0.50) during the sample collection period. Similarly, we found no difference in left brain weights (% of body weight; site of HI injury) between HI-treated and sham mice (0.02%, p = 0.74). For longer term studies, changes in brain weight may need to be considered, potentially by incorporating a time-dependent variable into our brain PK model. Second, our findings are limited to animal HI brain injury models. In humans, perinatal hypoxic-ischemic injury in infants not only causes brain injury but also affects other drug-eliminating organs such as the liver and kidneys (Razif et al., 2024). Although we did not evaluate the impact of extra-neurological injury on the PK of 7,8-DHF, our results on 7,8-DHF brain distribution provide crucial insights into the differences between adult and neonatal mice. For example, the low extent of 7,8-DHF brain distribution in neonatal mice should be considered when selecting dosing regimens for future studies aimed at establishing brain exposure-response or exposure-toxicity relationships (i.e., pharmacodynamics). Future studies should also consider the impact of extra-neurological injury such as renal or hepatic impairment on the PK of 7,8-DHF. Third, since therapeutic hypothermia is the standard of care in infants with HIE in high resource settings, future studies evaluating the PK and pharmacologic effects of 7,8-DHF under hypothermic conditions are warranted with close monitoring of the core temperatures during and after the HI induction. Interestingly, in rats with HI-related brain injury, the neuroprotective effects of TH were found to be significant in females but not in males (Wood TR. et al., 2020). Therefore, the sex-specific effects of 7,8-DHF under hypothermic conditions may be convoluted and require careful investigation.

Ultimately, the understanding of systemic and brain PK of 7,8-DHF among neonatal mice could be applied to larger animal models of neonatal HIE especially the ones that show the kidney and liver injury phenotype that is not present in Vannucci’s neonatal rodent model of HI. Together with the pharmacodynamic data, this PK knowledge may help in selecting dosing regimens needed to achieve target exposures that maximize efficacy and minimize toxicity, advancing the translation of 7,8-DHF therapy from preclinical to clinical studies.

## 5 Conclusion

The plasma and brain PK of 7,8-DHF in neonatal mice were similar between males and females. The sex-biased neuroprotective effects of 7,8-DHF observed in our previous studies are likely due to pharmacodynamic differences rather than PK. Developmental changes in neonatal mice and different routes of drug administration may explain the differences in PK profiles observed in our study compared to previously reported PK profiles in adult mice. Furthermore, the low extent of 7,8-DHF brain distribution and the potential effects of the active metabolites should be considered in future studies evaluating the therapeutic effects of 7,8-DHF.

## Data Availability

The data presented in the study are deposited in the Dryad repository, DOI: 10.5061/dryad.fbg79cp5q.
