# Supplementary material for: Pharmacokinetics of 7,8-dihydroxyflavone in neonatal mice with hypoxia-ischemia related brain injury
Source: Front Pharmacol. 2025 Jan 15;15:1508696. doi: 10.3389/fphar.2024.1508696 (PMC11775835; doi:10.3389/fphar.2024.1508696)
Supplement: Supplementary file 1 [file Supplementaryfile1.docx]

Supplementary Material

Pharmacokinetics of 7,8-Dihydroxyflavone in Neonatal Mice with Hypoxia-Ischemia Related Brain Injury

Running title: 7,8-Dihydroxyflavone pharmacokinetics in neonatal mice

Sin Yin Lim^1*^, Cameron Scarlett^2^, Sefer Yapici^3^, Peter Ferrazzano^3,4^, Pelin Cengiz^3,4^

^1^Pharmacy Practice and Translational Research Division, School of Pharmacy, University of Wisconsin-Madison, Madison, Wisconsin, USA

^2^Analytical Instrumentation Center, School of Pharmacy, University of Wisconsin-Madison, Madison, Wisconsin, USA

^3^Waisman Center, School of Medicine and Public Health, University of Wisconsin-Madison, Madison, Wisconsin, USA

^4^Department of Pediatrics, School of Medicine and Public Health, University of Wisconsin-Madison, Madison, Wisconsin, USA

***Correspondence:**Sin Yin Lim
sinyin.lim@wisc.edu

# Pharmacokinetic Model Development

One-, two-, and three-compartment models were compared, and the base pharmacokinetic (PK) model was selected based on the Akaike information criterion (AIC), diagnostic plots, and parameter estimation uncertainty. The residual error model was chosen by comparing the AIC and diagnostic plots. AIC values for different models are reported in **Table S1**. The interindividual variability for each structural PK parameter (i.e., clearance, volume of distribution, distributional clearance, peripheral volume of distribution) was evaluated based on the estimated interindividual variability value, shrinkage, and objective function value (-2 log-likelihood, -2LL). If the removal of the interindividual variability terms with low estimates (<5%) or high shrinkage (>30%) had a minimal impact on -2LL, they were excluded from the model. Only the interindividual variability for clearance was included in the final model. The base model parameter estimates are reported in **Table S2**. A stepwise covariate search was used in the covariate analysis (**Table S3**). The threshold for forward covariate addition was a reduction of -2LL of 6.64 (p<0.01), and the threshold for backward covariate elimination was an increase of -2LL of 10.83 (p<0.001). The final model diagnostic plots for the plasma PK model are shown in **Figure S1**. A perfusion-limited distribution model was used to describe the PK of 7,8-DHF in the left and right brain hemispheres. The diagnostic plots for the brain model are shown in **Figure S2**. This physiologically based model fits well with the observed data; therefore, no other brain PK models were tested.

**Table S1**. Akaike information criterion values for various models

| **Model** | **AIC** |
| --- | --- |
| *Pharmacokinetic model* |  |
| 1-compartment | 969.9 |
| 2-compartment | 807.3 |
| 3-compartment | 808.6 |
|  |  |
| *Error Model* |  |
| Proportional | 807.3 |
| Additive | 1040 |

**Table S2**. Pharmacokinetic parameter estimates of the base model.

| **Parameter^a^** | **Population Mean (RSE)** |
| --- | --- |
| V_1_ (mL/kg) | 1517 (59.3) |
| CL (mL/min/kg) | 235.3 (21.6) |
| V_2_ (mL/kg) | 5439 (43.1) |
| CL_d_ (mL/min/kg) | 86.17 (37.5) |
| Proportional error (%) | 5 (fixed) |
| IIV_CL_ (%) | 23.3 (20.1) |

^a^Parameters are “apparent” values as the bioavailability of intraperitoneal 7,8-DHF injection is unknown.

V_1_, central volume of distribution; CL, clearance; V_2_, peripheral volume of distribution; CL_d_, distributional clearance; IIV, interindividual variability; RSE, relative standard error in percentage

**Table S3**. Covariate analysis.

| **Model**^a^ | **-2LL** | **Δ-2LL** | **Action** |
| --- | --- | --- | --- |
| Forward addition |  |  |  |
| *Step 1* |  |  |  |
| CL (no covariate) | 797.88 |  |  |
| CL-surgery | 772.15 | -25.73^b^ | Surgery added |
| CL-sex | 796.24 | -1.64 |  |
| *Step 2* |  |  |  |
| CL-surgery | 772.15 |  |  |
| CL-surgery-sex | 770.32 | -1.83 | Sex not added |
| Backward elimination |  |  |  |
| *Step 1* |  |  |  |
| CL-surgery | 772.15 |  |  |
| CL (no covariate) | 797.88 | +25.73^c^ | Surgery retained |

^a^Surgery refers to sham or hypoxic-ischemic treatment.

^b^Reduction of -2LL >6.64, corresponding to p-value of <0.01

^c^Increase of -2LL >10.83, corresponding to p-value of <0.001

-2LL, -2 log likelihood; Δ-2LL, change of -2LL


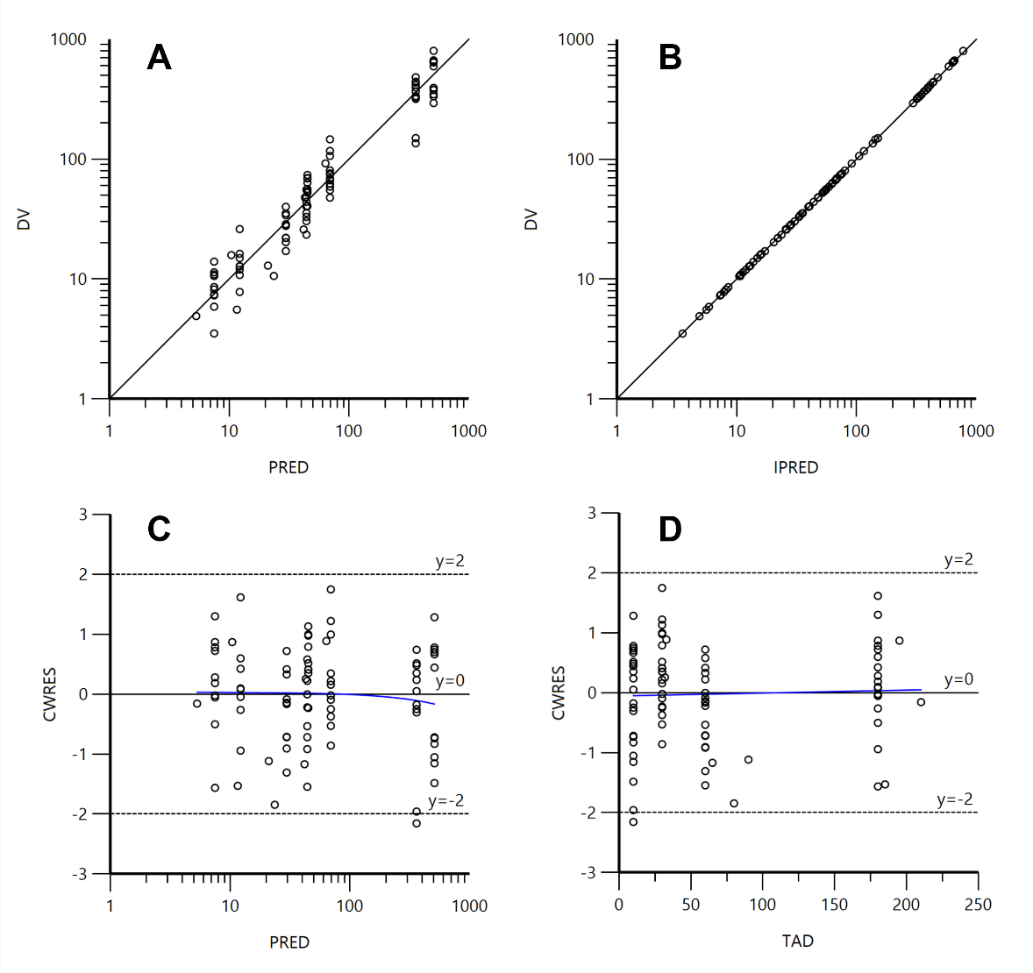


**Figure S1**. Diagnostic plots of the final plasma pharmacokinetic model. (A) Observed (DV) versus population prediction (PRED) of plasma concentration, (B) observed versus individual prediction (IPRED) of plasma concentration, (C) conditional weighted residual (CWRES) versus population prediction, and (D) CWRES versus time after dose (TAD).


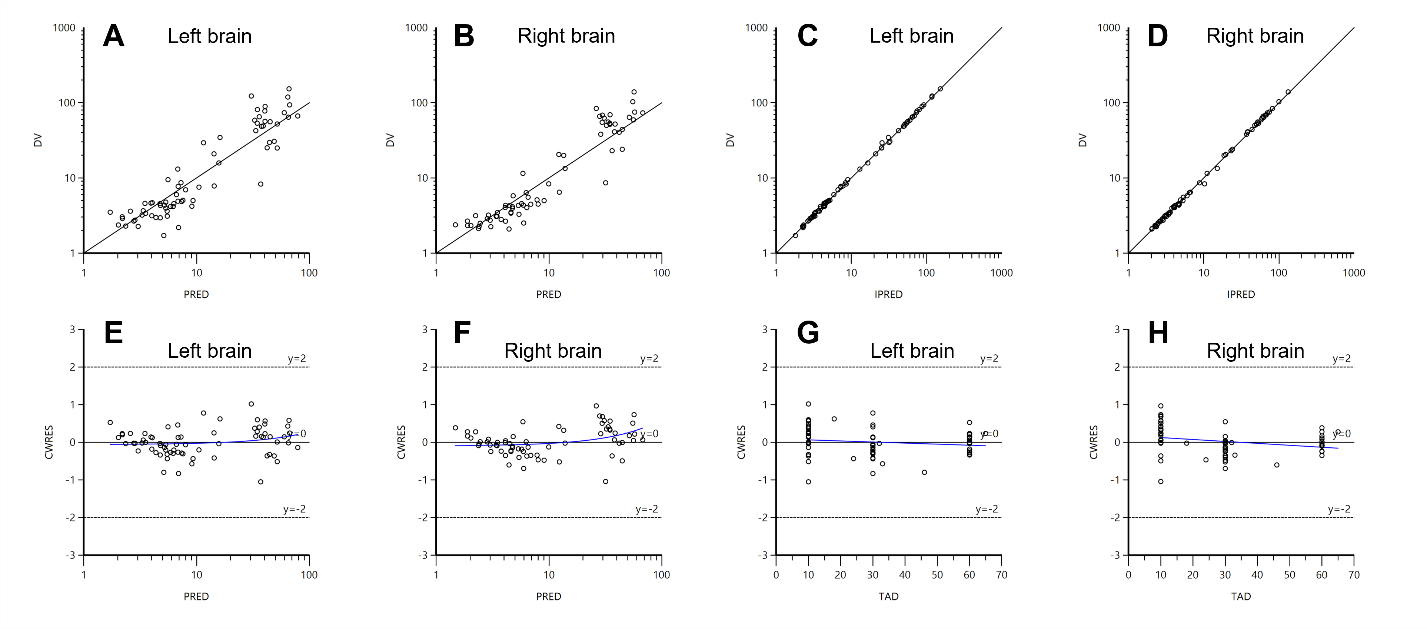


**Figure S2**. Diagnostic plots of the final brain pharmacokinetic model. (A-B) Observed (DV) versus population prediction (PRED) of left and right brain concentration, (C-D) observed versus individual prediction (IPRED) of left and right brain concentration, (E-F) conditional weighted residual (CWRES) versus population prediction of left and right brain concentration, and (D) CWRES versus time after dose (TAD) of left and right brain concentration.

# Impact of Fixed Intraindividual Variability Values on Pharmacokinetic Parameter Estimation

Because only one sample was collected from each animal, the intraindividual variability is not discernible from the interindividual variability. To estimate covariate effects on the interindividual variability of PK parameters, the intraindividual variability (residual error) was fixed at 5% for plasma concentrations and 10% for brain concentrations, based on the bioanalytical variability of the quality control samples. It should be noted that the true intraindividual variability is unknown. In two previous studies that evaluated the single sample per subject design, it was found that when the true intraindividual variability is 10-15%, varying the fixed intraindividual variability from very small to 30% did not significantly impact the PK parameter estimation.^1,2^

We compared various levels of fixed intraindividual variability (ranging from 5% to 50%; **Table S4**). These changes had little impact on the estimated interindividual variability (ranging from 20% to 14%). We observed that when the intraindividual variability was relatively higher (>20%), the shrinkage of clearance interindividual variability also increased (>30%). This suggests that if the true intraindividual variability is high (>20%), the estimated clearance value for each individual may become biased towards the population average value. Additionally, the estimated PK parameters were found to be relatively similar when the intraindividual variability values were within 20%. This suggests that the single sample per subject design may rely on a true intraindividual variability that is relatively low (<20%) in order to obtain reliable PK parameters, interindividual variabilities, and covariate effects.

**Table S4**. Impact of varying intraindividual variability (error%) on the parameter estimation.

| **Error%** | **-2(LL)** | **AIC** | **CL (RSE)** | **V_1_ (RSE)** | **CL_d_ (RSE)** | **V_2_ (RSE)** | **IIV%** | **shrinkage** |
| --- | --- | --- | --- | --- | --- | --- | --- | --- |
| 5% | 772.15 | 784.15 | 270.15 (18.01) | 1592.13 (49.20) | 90.53 (29.59) | 5802.85 (35.17) | 20.26 | 0.049 |
| 10% | 773.23 | 785.23 | 279.35 (23.30) | 1742.96 (65.92) | 96.52 (37.52) | 6209.39 (46.39) | 19.71 | 0.076 |
| 15% | 772.91 | 784.91 | 304.73 (11.62) | 2246.88 (38.78) | 111.48 (16.64) | 7412.30 (25.65) | 18.44 | 0.117 |
| 20% | 767.12 | 779.12 | 326.34 (6.69) | 2991.28 (19.37) | 122.61 (12.92) | 8872.56 (16.93) | 14.26 | 0.283 |
| 25% | 760.90 | 772.90 | 329.92 (5.96) | 3214.34 (14.00) | 125.82 (11.76) | 9473.33 (14.27) | 10.41 | 0.480 |
| 50% | 783.15 | 795.15 | 375.56 (-) | 3796.86 (-) | 148.90 (-) | 11634.54 (-) | 0.00 | 1.000 |

V_1_, central volume of distribution in mL/kg; CL, clearance in mL/min/kg; V_2_, peripheral volume of distribution in mL/kg; CL_d_, distributional clearance in mL/min/kg; IIV, interindividual variability; RSE, relative standard error in percentage; -, not available

**References**

1. Ette, E.I., et al., *Analysis of animal pharmacokinetic data: performance of the one point per animal design.* J Pharmacokinet Biopharm, 1995. **23**(6): p. 551-66.

2. Hing, J.P., et al., *Is mixed effects modeling or naïve pooled data analysis preferred for the interpretation of single sample per subject toxicokinetic data?* J Pharmacokinet Pharmacodyn, 2001. **28**(2): p. 193-210.
